# Supplementary material for: Primary cilia and SHH signaling impairments in human and mouse models of Parkinson’s disease
Source: Nat Commun. 2022 Aug 16;13:4819. doi: 10.1038/s41467-022-32229-9 (PMC9380673; doi:10.1038/s41467-022-32229-9)
Supplement: Supplementary file 1 — Supplementary Information [file 41467_2022_32229_MOESM1_ESM.pdf]

Supplementary Information File

Primary cilia and SHH signaling impairments in human and mouse models of Parkinson's disease

Authors: Sebastian Schmidt[1,2]†, Malte D. Luecken[3]†, Dietrich Trümbach[1,4]†, Sina Hembach[1,2], Kristina M. Niedermeier[1,2], Nicole Wenck[1,2], Klaus Pflügler[1,2], Constantin Stautner[1,2], Anika Böttcher[5], Heiko Lickert[5], Ciro Ramirez-Suastegui[3], Ruhel Ahmad[6], Michael J. Ziller[7], Julia C. Fitzgerald[8], Viktoria Ruf[9,10], Wilma D.J. van de Berg[11], Allert J. Jonker[11], Thomas Gasser[8], Beate Winner[12], Jürgen Winkler[13], Daniela M. Vogt Weisenhorn[1,2], Florian Giesert[1]*, Fabian J. Theis[3,14]*, Wolfgang Wurst[1,2,10,15]*

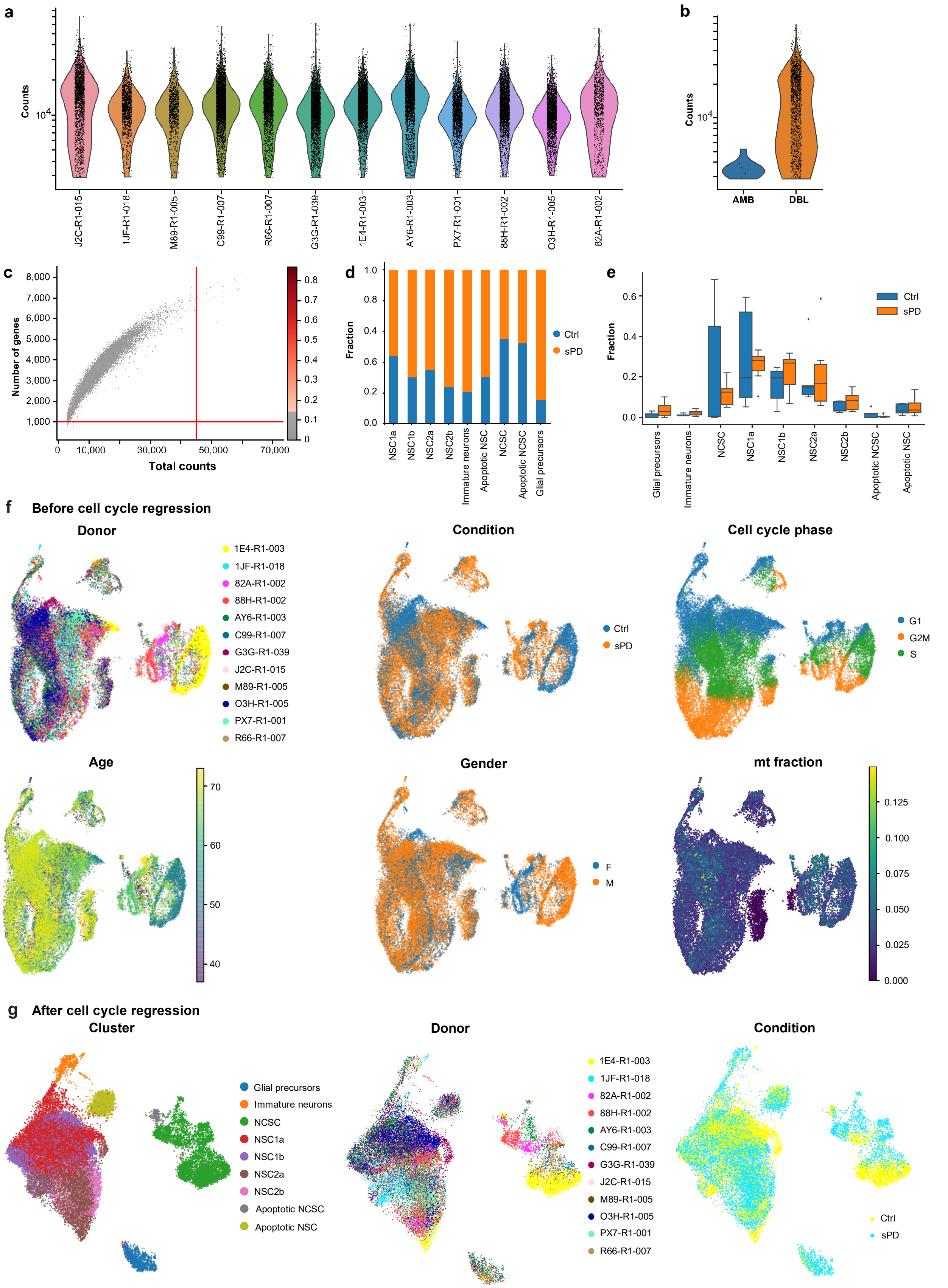

Supplementary Fig. 1 | scRNA-seq quality control and metadata visualization. (a) Distribution of unique molecular identifier (UMI) counts per donor showing consistent

quality of cells. **(b)** Distribution of UMI counts for cells that could not be assigned to a donor by demuxlet (AMB) and cells that were called as doublets (DBL). **(c)** Plot of number of detected genes per cell vs number of UMI counts per cell with implemented quality control thresholds shown as red lines. We filtered the data to have a minimum of 1,000 genes per cell at maximum 45,000 total counts. This also eliminates most cells with a high fraction of mitochondrial reads. **(d)** Contribution of Ctrl and sPD patients to clusters. **(e)** Box plot of cell type proportions per patient divided by disease condition as generated by scCODA. No significant differences were found for any cell type. **(f)** UMAP visualizations of processed scRNA-seq dataset with metadata annotations for donor, disease condition (Ctrl, sPD), cell cycle phase (G1-, G2/M-, S-phase), donor age, gender (male: M, female: F) and fraction of UMI counts from mitochondrial (mt) genes. **(g)** UMAP visualization of processed scRNA-seq dataset with cell cycle effects regressed out. Cells are colored by cell identity clusters, donor and disease condition (Ctrl, sPD). n = 30,557 hNPCs derived from 5 Ctrl and 7 sPD clones.

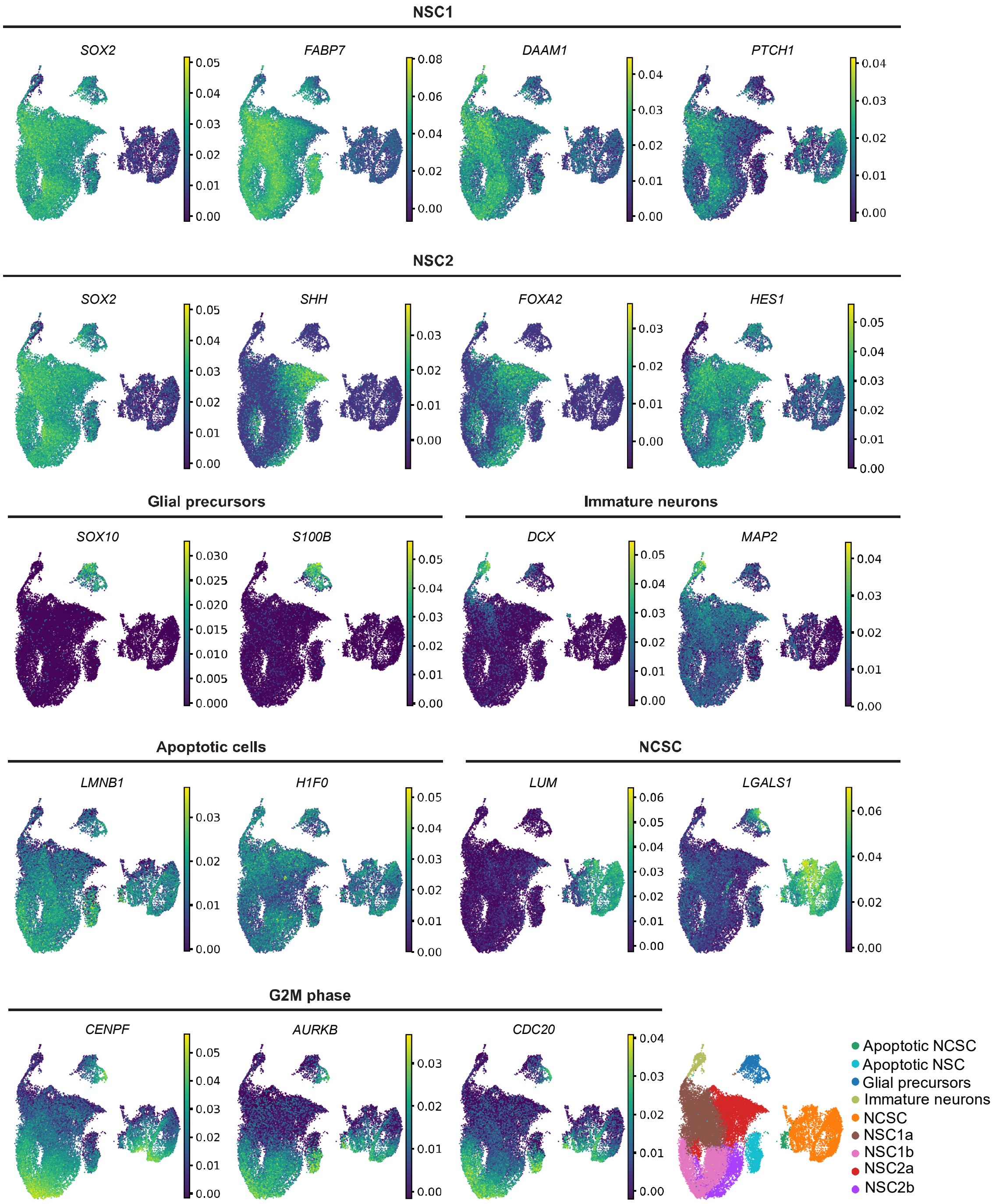

Supplementary Fig. 2 | scRNA-seq cluster annotation. UMAP visualization of MNN-corrected expression values of known cellular marker genes used for identifying clusters. **NSC1:** *SOX2*, *DAAM1*, *FABP7*, *PTCH1*, *DLL1*; **NSC2:** *SOX2*, *SHH*, *FOXA2*, *HES1*; **Glial precursors:** *SOX10*, *S100B*; **immature neurons:** *DCX*, *MAP2*; **apoptotic cells:** *LMNB1*, *H1F0*; *NCSC*: *LUM*, *LGALS1*; **NSC1/2 G2M:** *CENPF*, *AURKB*, *CDC20*. Final cluster annotations are shown in the UMAP plot in the bottom right corner. n = 30,557 hNPCs derived from 5 Ctrl and 7 sPD clones.

a

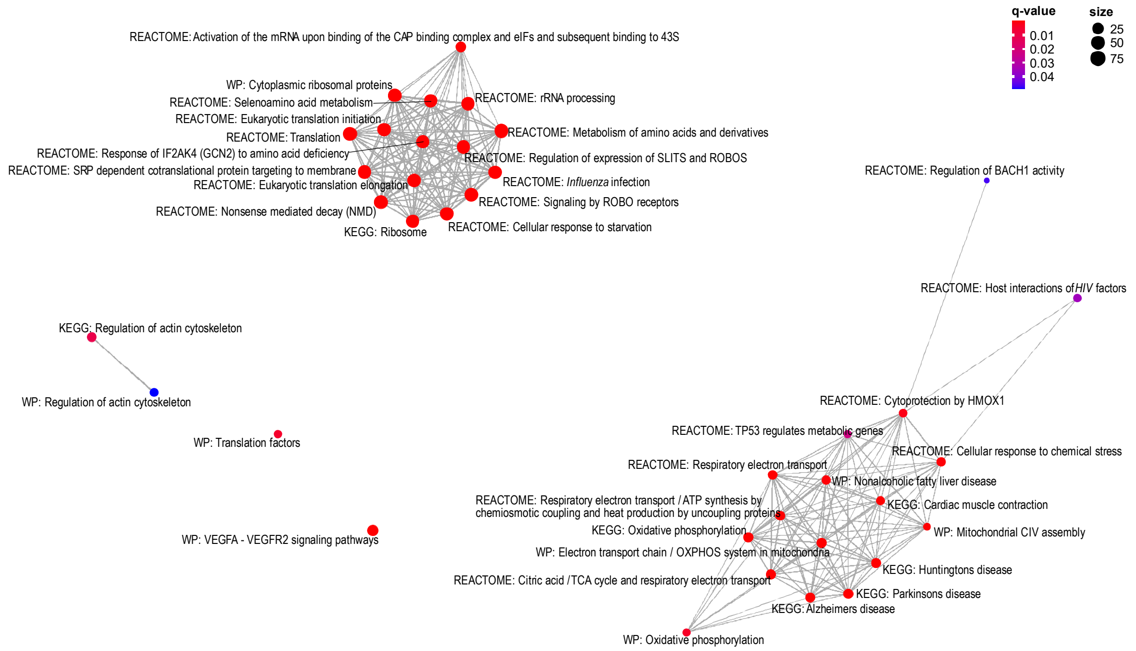

b

Differentially expressed genes Ctrl vs sPD	Number of genes, q < 0.05	Number of protein coding genes, q < 0.05	Number of upregulated genes, q < 0.05	Number of downregulated genes, q < 0.05	Number of genes, | FC | > 20 %, q < 0.01	Number of upregulated genes, | FC | > 20 %, q < 0.01	Number of downregulated genes, | FC | > 20 %, q < 0.01
NSC1a	4467	3137	491	3976	1004	357	647
NSC2a	4348	3170	479	3869	1286	242	1044
NSC1b	4997	3543	548	4449	1266	434	832
NSC2b	2275	1593	340	1935	758	265	493
Apoptotic NSC	593	435	169	424	382	157	225
Glial precursors	4417	3221	3891	526	3951	3611	340
Immature neurons	599	396	277	322	439	259	180
NCSC	3917	2243	3711	206	3322	3202	120
Apoptotic NCSC	619	458	290	329	546	262	284

c

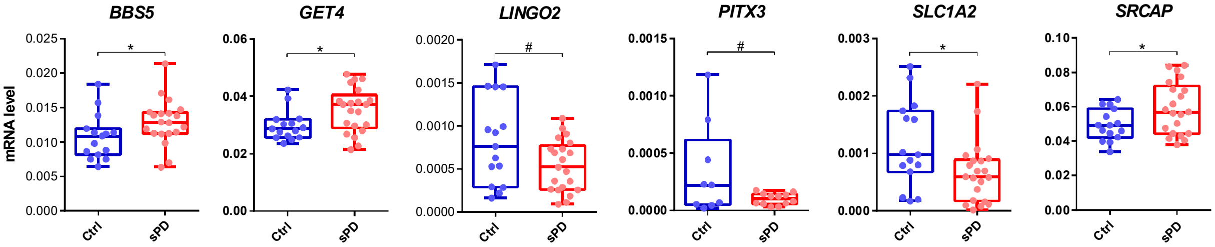

Supplementary Fig. 3 | Expression values and validation of top DEGs. (a) Network of enriched canonical pathways for DEGs of the root population with edges weighted by the ratio of overlapping gene sets. The network can be subdivided into functional modules associated with 'mitochondria and disease' and 'translation'. FDR corrected p-values are represented by q-values. **(b)** Number of DEGs per cluster by negative binomial model fit and wald test over the condition covariate as implemented in diffxpy. **(c)** RT-qPCR validation of DEGs determined by scRNA-seq. Target gene mRNA levels are normalized to BestKeeper[70] calculated from *GAPDH*, *HPRT1* and *ACTB.* n = 15 Ctrl and 21 sPD samples from 3 independent differentiations of 5 Ctrl and 7 sPD clones. Boxplots display the median and range from the 25th to 75th percentile.

Whiskers extend from the min to max value. P values are determined by two-sided *t*-test *PITX3* (p=0.0541), *SRCAP* (p=0.0321); two-sided Welch-test *LINGO2* (p=0.0955), *SLC1A2* (p=0.0255); two-sided Mann-Whitney-U test *BBS5* (p=0.0392), *GET4* (p=0.0358). #, p < 0.1; *, p < 0.05; **, p < 0.01; ***, p < 0.001. Scale bars = 10 µm. Source data are provided as a Source Data file.

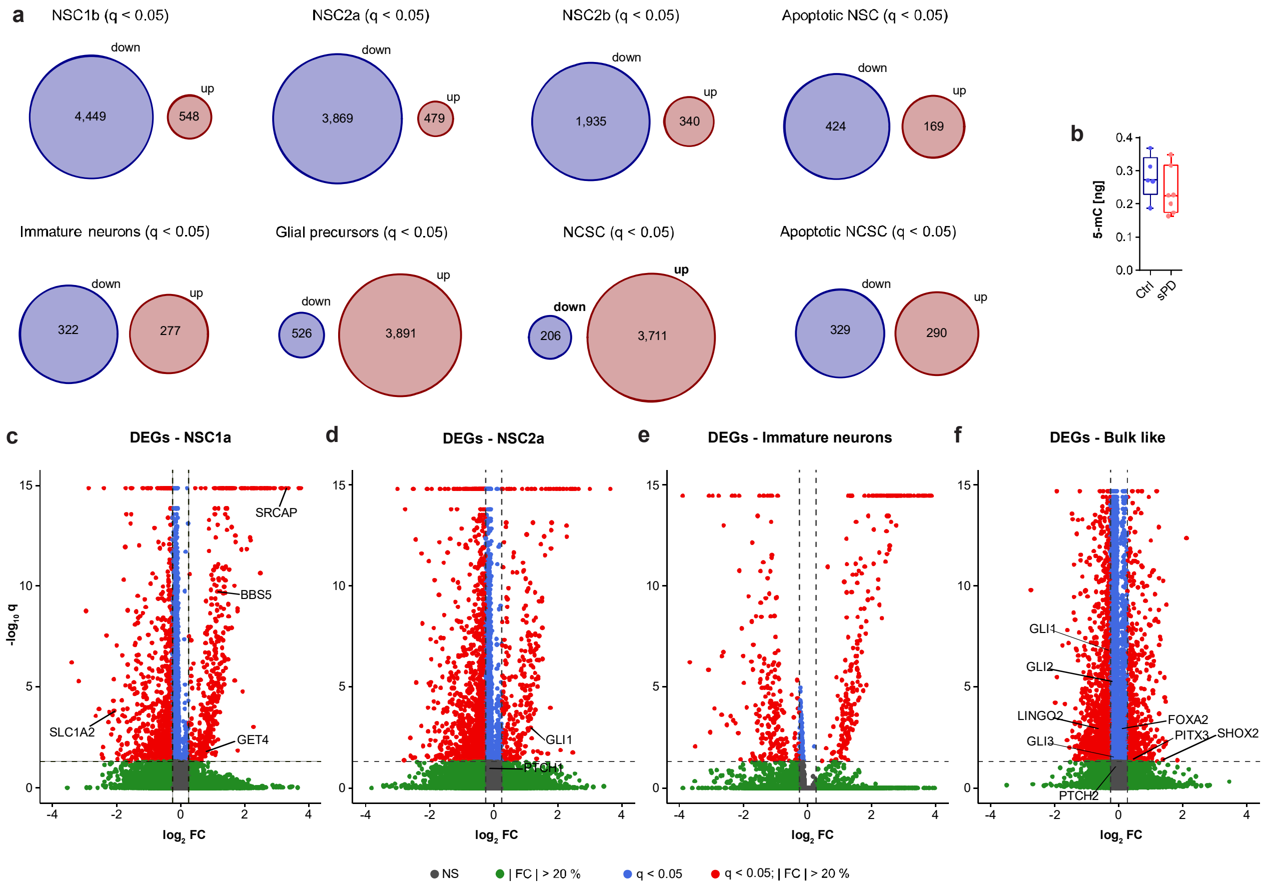

Supplementary Fig. 4 | Cluster specific gene expression changes in sPD pathology. (a) Number of up- and downregulated DEGs in all clusters displayed as Venn diagrams. Diagrams for NSC1a are shown in **Fig. 4b**. FC, fold change. **(b)** Analysis of DNA methylation levels (5-methylcytosine (5-mC) levels in [ng]) in hNPC clones. n = 5 Ctrl and 7 sPD clones, in triplicates. Boxplot is displayed from min to max values with all data points shown. P value is determined by two-sided *t*-test. **(c) – (f)** Volcano plots showing significances and fold changes (FC) for DEGs of clusters NSC1a, NSC2a, immature neurons and the bulk like state, respectively. Highlighted are genes independently validated by RT-qPCR **(Fig. 6b; Supplementary Fig. 3c).** Boxplots display the median and range from the 25th to 75th percentile. Whiskers extend from the min to max value. Each dot represents one patient. Source data are provided as a Source Data file.

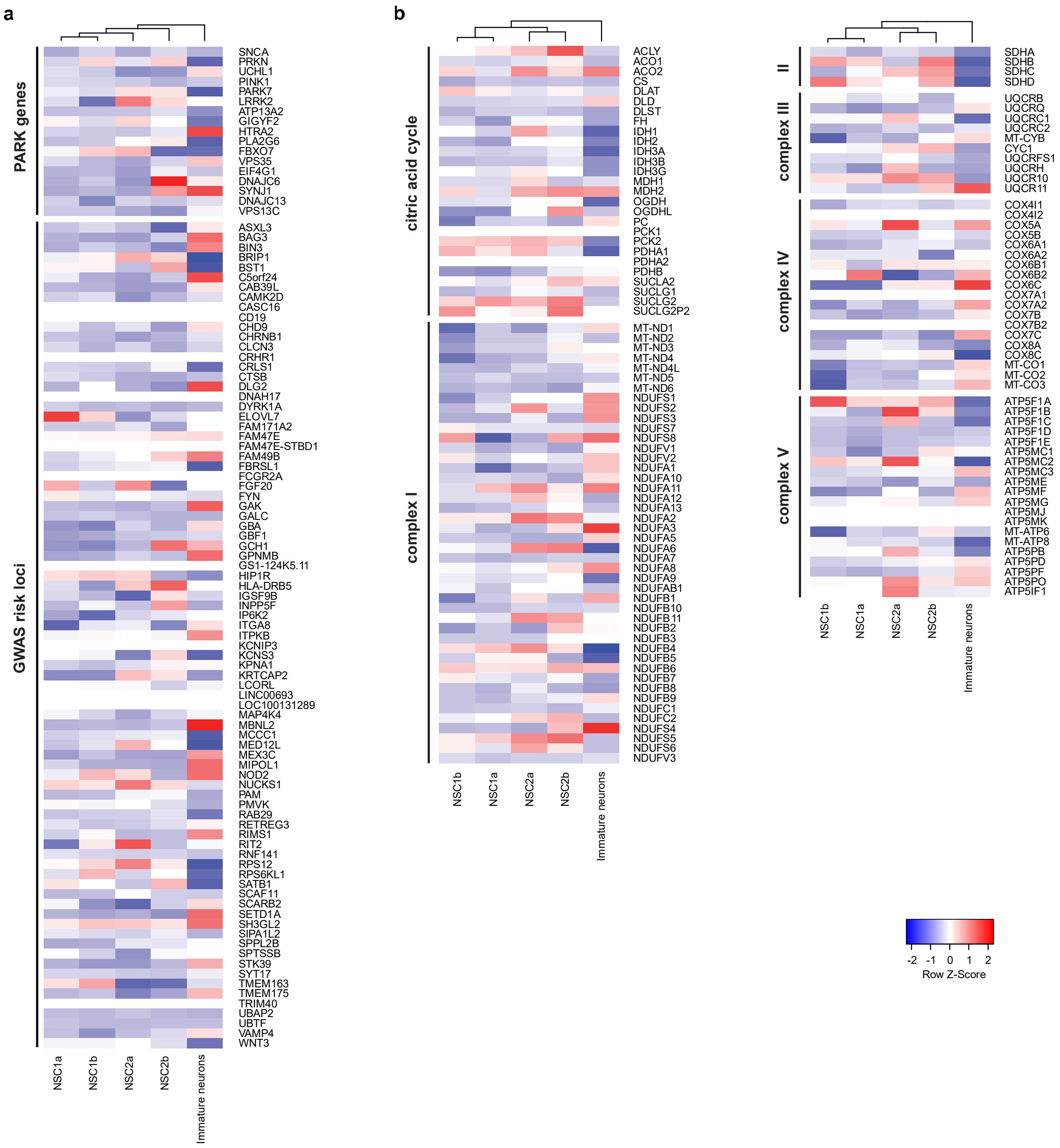

Supplementary Fig. 5 | Heatmaps visualizing expression changes of PD and mitochondria associated genes over clusters. (a) Heatmap showing log2 transformed fold changes (FC) with columns scaled by z-score for PD associated and **(b)** citric acid cycle / electron transport chain associated genes. Hierarchical clustering of cell types represented by the dendrogram reveals their similarity across DEGs.

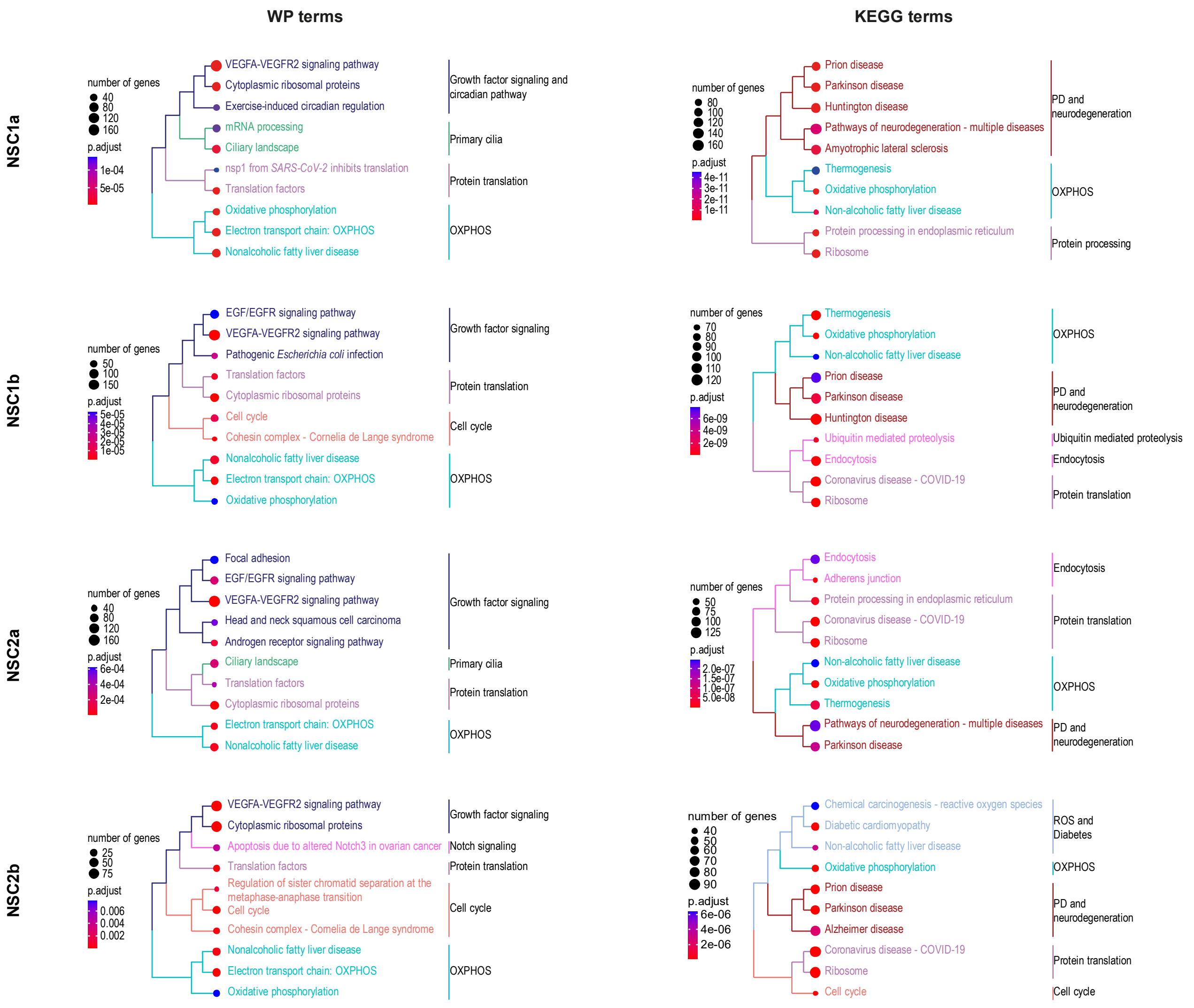

Supplementary Fig. 6 | Pathway enrichment analysis. Enriched KEGG and WikiPathway (WP) terms for the DEGs of the NSC clusters (q < 0.05). P values were determined by one-sided hypergeometric tests. p-values corrected for multiplicity are represented by q-values.

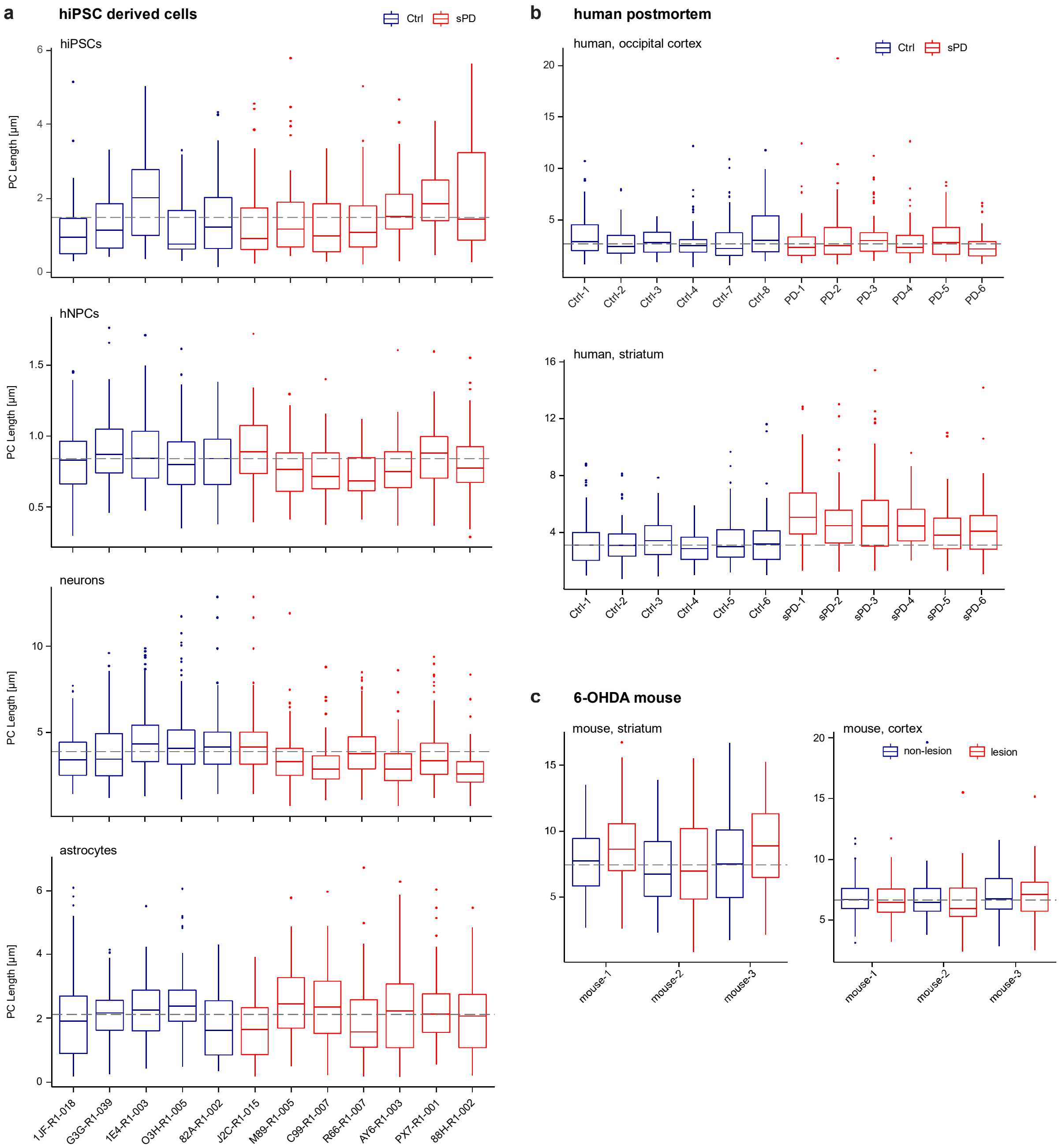

Supplementary Fig. 7 | Average PC length in sPD. (a) PC length (in µm) analyzed in sPD and Ctrl hiPSCs (n = 37-79 cilia per clone), hNPCs (n = 74-123 cilia per clone), neurons (n = 131-385 cilia per clone) and astrocytes (n = 100 cilia per clone). PC length was measured in immunostainings with anti-ARL13B and anti-SOX2 (hiPSCs) or anti-NES (hNPCs) or anti-TUBB3 (neurons) or anti-GFAP (astrocytes). Dotted lines indicate the median PC length of Ctrl clones. Experiments were performed in triplicates, n = 5 Ctrl and 7 sPD clones. **(b)** PC length (in µm) analyzed in human occipital cortex (n = 75-105 cilia per individual) and striatum (n = 79-133 cilia per individual). PC length was measured in paraffin embedded brain sections immunostained with anti-ARL13B. Dotted lines indicate the median PC length of Ctrl clones. Brain sections were obtained from 6 PD patients and 6 Ctrls. **(c)** PC length (in µm) analyzed in mouse dorsal striatum (n = 87-115 cilia per mouse) and cortex (n = 71-111 cilia per mouse).

PC length was measured in 40 µm free floating brain sections of 3 6-OHDA injected mice immunostained with anti-Adcy3 and anti-Rbfox3. Dotted lines indicate the median PC length of the non-lesion sides. Boxplots display the median and range from the 25th to 75th percentile. Whiskers extend to the min or max value, no further than 1.5 * inter-quartile range. Outlying points are plotted individually. Source data are provided as a Source Data file.

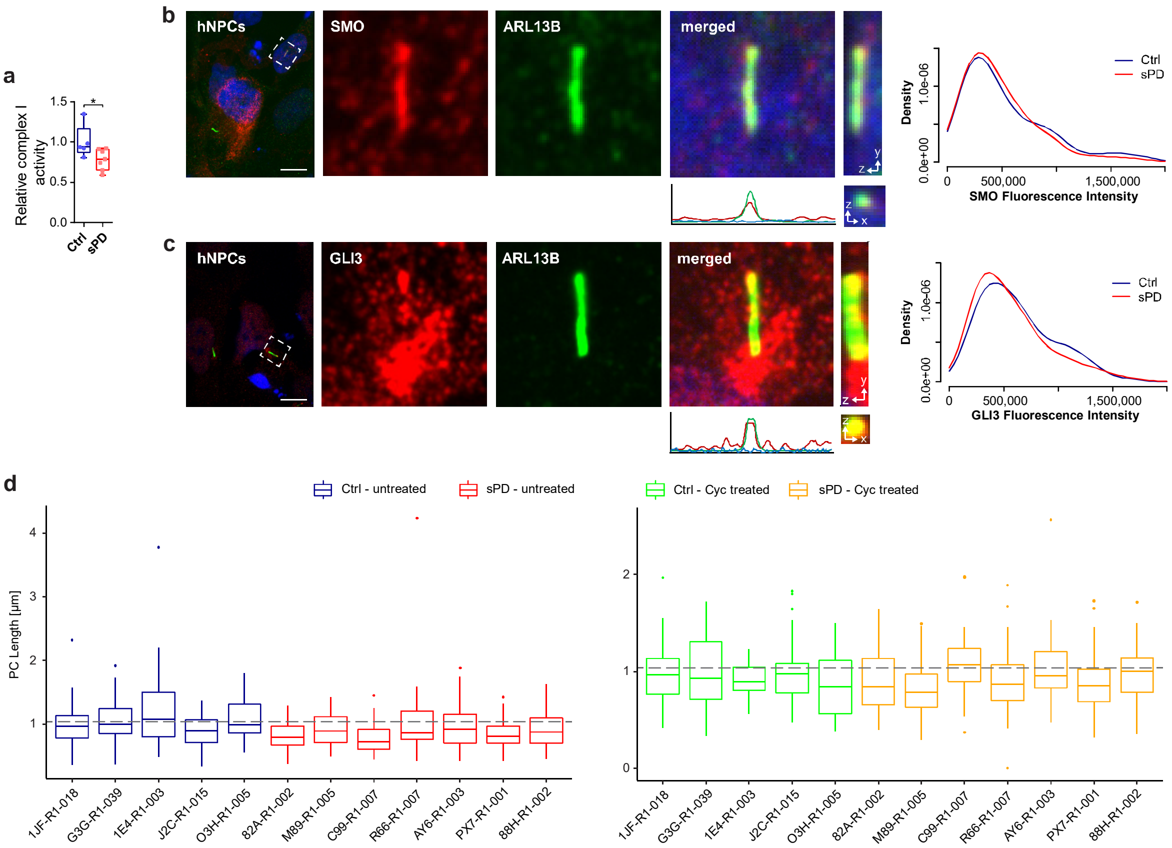

Supplementary Fig. 8 | Cell proliferation rate and ciliary GLI3, SMO levels in hNPCs. (a) Analysis of relative Complex I activity in hNPC clones normalized to Ctrl levels. Boxplots display the median and range from the 25th to 75th percentile. Whiskers extend from the min to max value. Each dot represents one patient. **(b)** Ciliary SMO protein levels analyzed in hNPCs by immunostaining with anti-SMO and anti-ARL13B followed by quantitative imaging of z-stacks. Immunostainings are exemplarily shown for iG3G-R1-039. n > 250 PC per condition. **(c)** Ciliary GLI3 protein levels analyzed in hNPCs by immunostaining with anti-GLI3 and anti-ARL13B followed by quantitative imaging of z-stacks. n > 250 PC per condition. PC immunostaining is exemplarily shown for iG3G-R1-039. **(d)** PC length (in µm) was analyzed in hNPCs (DMSO ctrl and Cyc treated - 10 µM for 4 days) immunostained with anti-ARL13B and anti-NES. Dotted lines indicate the median PC length of Ctrl clones (DMSO ctrl). n > 100 PC per clone. All experiments were performed in triplicates, n = 5 Ctrl and 7 sPD clones. Boxplots display the median and range from the 25th to 75th percentile. Whiskers extend to the min or max value, no further than 1.5 * inter-quartile range. Outlying points are plotted individually. P values are determined by two-sided *t*-test **a** (p=0.0484); two-sided Kolmogorov-Smirnov test **b**, **c**. *, p < 0.05; **, p < 0.01; ***, p < 0.001. Scale bars = 10 µm. Source data are provided as a Source Data file.

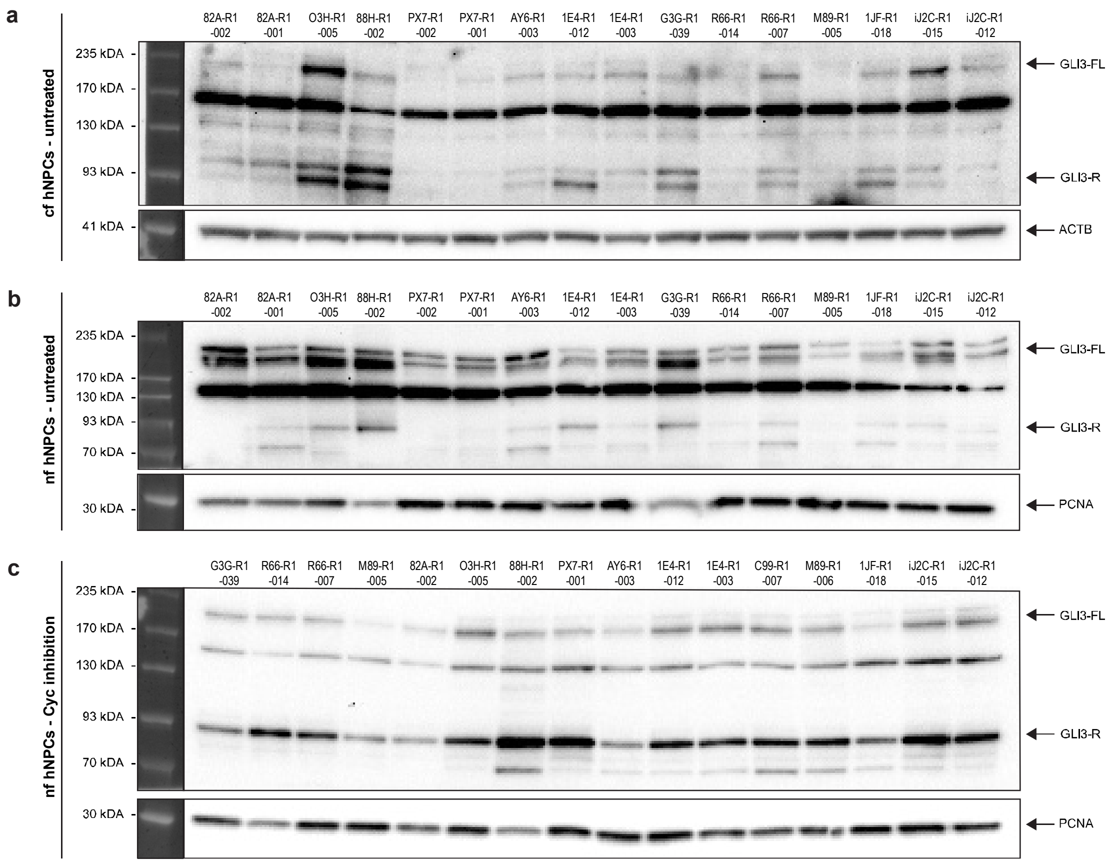

Supplementary Fig. 9 | GLI3 processing in hNPCs. (a) Western blot shown exemplarily for the cytoplasmic and **(b)** nuclear fraction (NF) isolated from untreated and **(c)** Cyc treated (10 µM for 4 days) hNPCs. The nf of proteins were immunoblotted using antibodies against GLI3 and PCNA, the cf of proteins were immunoblotted using antibodies against GLI3 and ACTB. All experiments were performed in triplicates, n = 5 Ctrl and 7 sPD individuals. Source data are provided as a Source Data file.

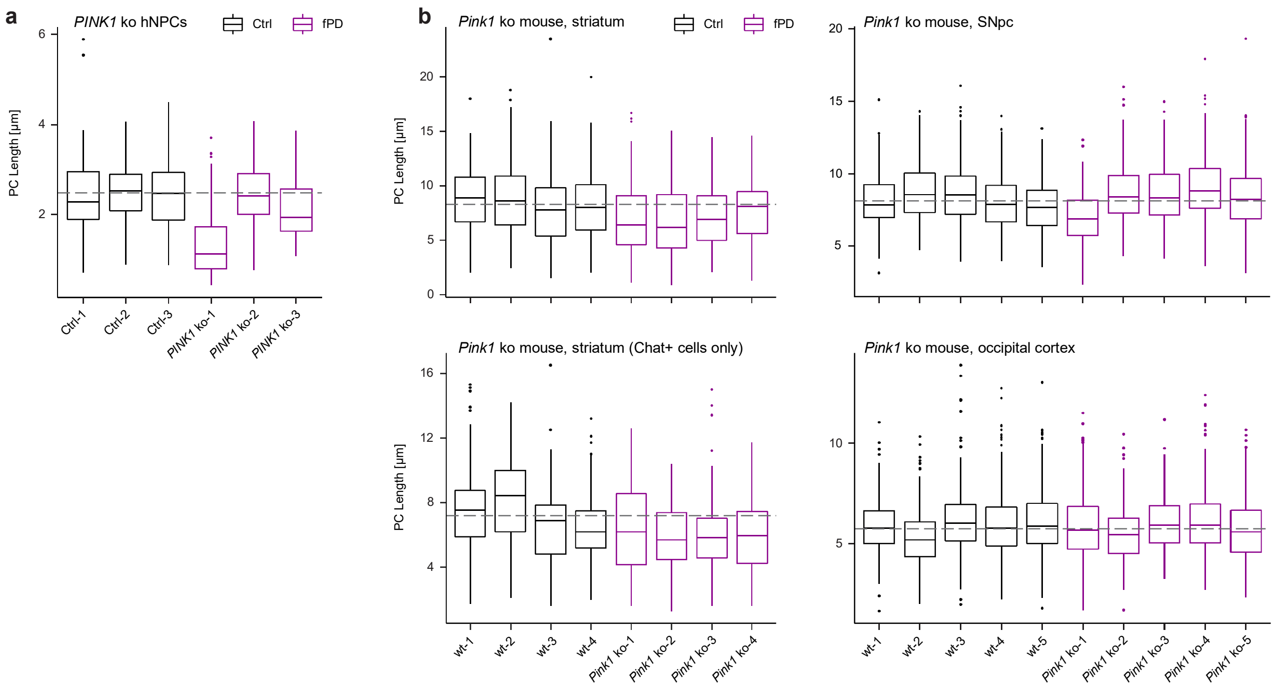

Supplementary Fig. 10 | Average PC length in fPD. (a) PC length (in µm) analyzed in *PINK1* ko and isogenic Ctrl hNPCs with n > 90 PC per condition from three independent differentiations. Dotted lines indicate the median PC length of Ctrl clones. **(b)** PC length (in µm) analyzed in mouse dorsal striatum, SNpc and occipital cortex. PC length was measured in 40 µm free floating brain sections from 4 *Pink1* ko and 5 wt mice immunostained with anti-Adcy3 and anti-Rbfox3 (all neurons) or anti-Chat (cholinergic neurons). Dotted lines indicate the median PC length of Ctrl clones. n > 50 PC per mouse. Boxplots display the median and range from the 25th to 75th percentile. Whiskers extend to the min or max value, no further than 1.5 * inter-quartile range. Outlying points are plotted individually. Source data are provided as a Source Data file.

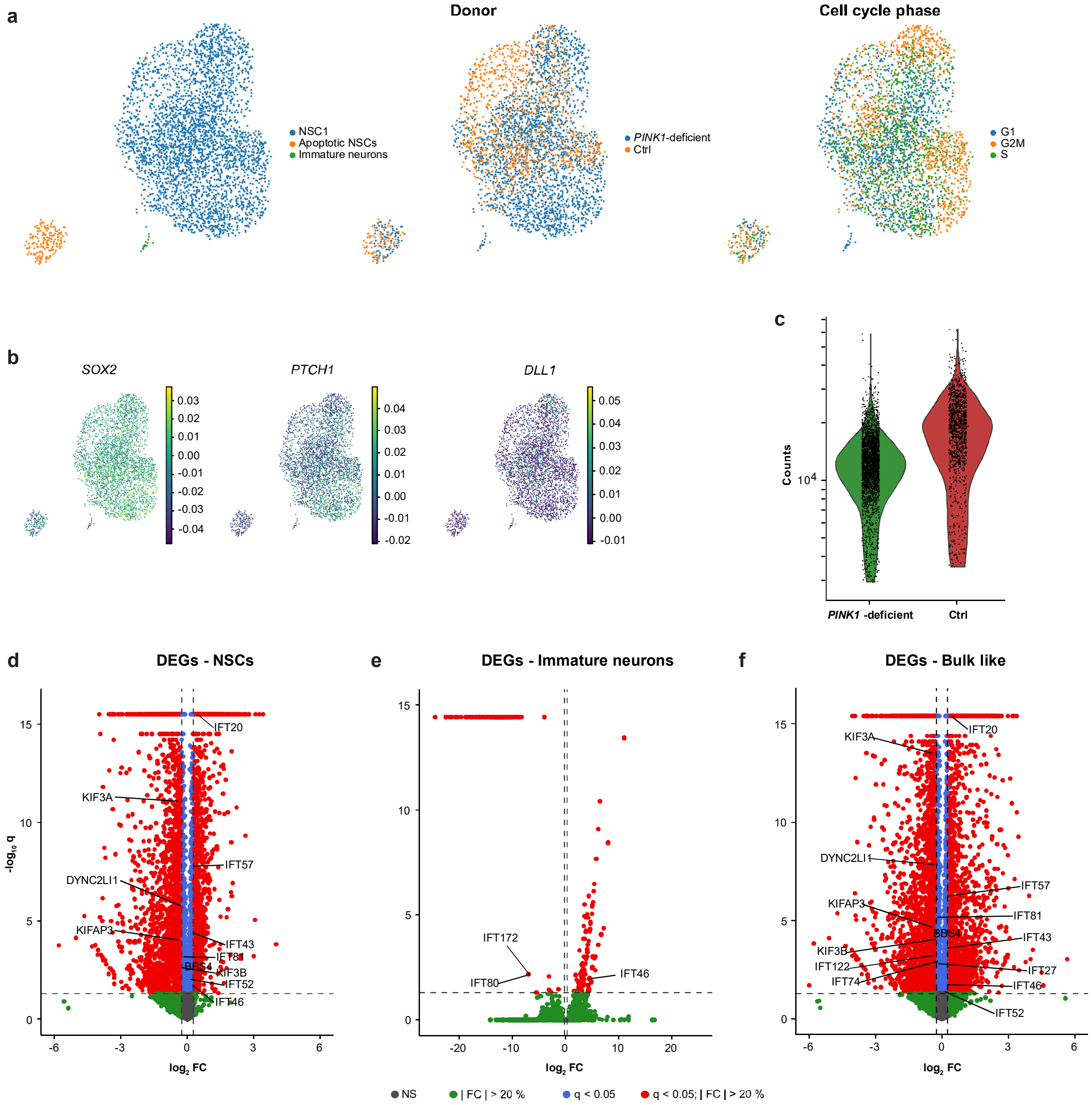

Supplementary Fig. 11 | scRNA-seq of *PINK1* ko hNPCs. (a) UMAP visualization of 3,671 annotated cells from a *PINK1* ko and isogenic Ctrl hNPC cell line for cell identity with metadata annotations for donor and cell cycle phase (G1-, G2/M-, S-phase). **(b)** UMAP visualization of MNN-corrected marker gene expression values for the genes *SOX2*, *PTCH1*, and *DLL1* that identify NSCs. **(c)** Distribution of UMI counts between *PINK1* ko and Ctrl populations indicating a higher cell sequencing depth in Ctrl cells. **(d) – (f)** Volcano plots showing significances and fold changes (FC) for DEGs of clusters NSC, immature neurons and the bulk like state, respectively. Highlighted are interesting genes of intraflagellar transport pathways.

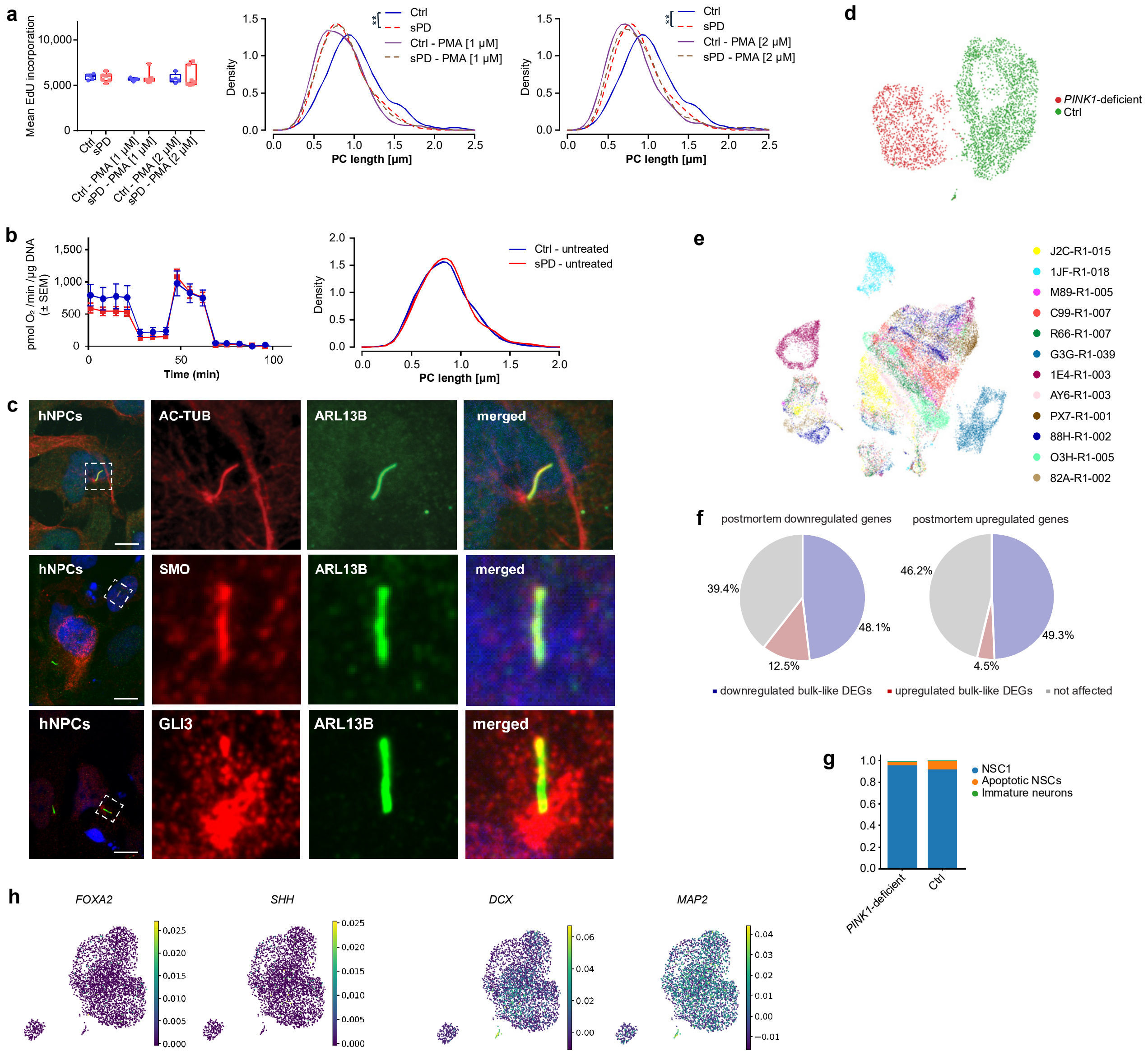

Supplementary Fig. 12 | Figures of rebuttal letter (in parts preliminary). (a) ReFig. 1 | (left) Cell proliferation rate of hNPCs (DMSO ctrl and PMA treated - 1 µM or 2 µM for 4 days) determined by EdU incorporation after 2 h of EdU treatment. Analyzed were n > 200 cells per individual and independent replicate. **(right)** Density plot illustrating the distribution of PC length (in µm) in an hNPC population (DMSO ctrl and PMA treated - 1 µM or 2 µM for 4 days). PC length was measured in immunostainings of hNPCs positive for anti-NES and anti-ARL13B. Analyzed were n >30 PC per individual and independent replicate. All experiments were performed in triplicates, n = 5 Ctrl and 7 sPD clones. **(b) ReFig. 2 | (left)** Mitochondrial stress test performed in hNPCs derived from 5 Ctrl and 7 sPD patients (in triplicates) 4 days after PMA removal using a Seahorse XFe96 Extracellular Flux Analyzer. Injected were (A) Oligomycin (1 µg/ml), (B) FCCP (0.5 µM) and (C) Rotenone (5 µM)/Antimycin A (2 µM). Measurement progression is shown with means ± standard error of the mean (SEM). **(right)** Density plot illustrating the distribution of PC length (in µm) in an hNPC population 4 days after PMA removal. PC length was measured in immunostainings of hNPCs positive for anti-NES and anti-

ARL13B. Analyzed were n >30 PC per individual and independent replicate. All experiments were performed in triplicates, n = 4 Ctrl and 7 sPD clones. **(c) ReFig. 3 |** Immunostaining with anti-ARL13B together with either **(upper)** anti-AC-TUB, **(middle)** anti-SMO, or **(lower)** anti-GLI3 using hNPCs. Immunostainings are exemplarily shown for iG3G-R1-039. **(d) ReFig. 5 |** UMAP plot of the fPD (*PINK1* ko) cell line data before MNN integration. **(e) ReFig. 4 |** UMAP plot of the sPD cell line data before MNN integration showing distinct donor structure in the embedding. **(f) ReFig. 6 |** Overlap of DEGs between the transcriptome analysis published by Wang et al., Nature Communications 2019 and the analysis described in this manuscript. Values indicate the percentage composition of DEGs identified by Wang et al. in our bulk-like DEGs. **(g) ReFig. 7 |** Stacked bar plot of cell type compositions per sample in fPD cell lines. The y-axis indicates the fraction of cells from a sample (N=2529 *PINK1*-deficient cells, N=1142 Isogenic Ctrl cells). **(h) ReFig. 8 |** UMAP plots of fPD line data colored by marker genes for NSC2 (*FOXA2*, *SHH*) and immature neurons (*DCX*, *MAP2*). N=3671 cells. P values were determined by two-sided *t*-test **a** (left), **b** (left); two-sided Kolmogorov-Smirnov test + linear mixed effects model **a** (middle, right), **b** (right). #, $p < 0.1$; *, $p < 0.05$; **, $p < 0.01$; ***, $p < 0.001$. Source data are provided as a Source Data file.

Supplementary Data 1 | Detailed description of retroviral-hiPSC clones received from the ForIPS consortium [10]. Column headings: "study_ID_sample" identifier for hiPSC clones in this study; "study_ID_individual" identifier for individuals in this study; "gender" biological gender of the respective individual; "age_biopsy_years" age in years of the respective individual at time of tissue biopsy; "years_of_illness" time in years between sPD diagnosis and tissue biopsy of the respective individual

Supplementary Data 2 | CNVs in hiPSC clones. CNVs identified for all hiPSC clones and genes (HGNC symbols) within these regions. Column headings: "State"; "Sample ID"; "Chromosome"; "Start [bp]"; "End [bp]"; "Size [bp]"; "Copy numbers"; "Genes within these regions"

Supplementary Data 3 | Pathways affected by CNVs. Enrichment analysis based on genes affected by CNVs in Ctrl or sPD hiPSC clones in curated pathways from the Pathway Studio Web (Elsevier). Enriched terms with $p < 0.05$ are shown for Ctrl or sPD clones. P values were determined by one-sided Fisher's exact tests. FDR corrected p-values are represented by q-values.

Column headings: "Name" denotes the annotation term; "Parent Folder" parent of the annotation term "Name" extracted from literature; "# of Entities" number of genes with a given annotation; "Expanded # of Entities" number of genes with a given annotation including associated members of functional protein classes; "Overlap" intersect of the number of genes from the input set with the number of genes from a given annotation ("Expanded # of Entities"); "Percent Overlap" proportion of overlapping genes from the input set in a given annotation ("Expanded # of Entities"); "Overlapping Entities" summarizes gene symbols from the input set with assigned annotation; "p-value" is the probability of overrepresentation calculated for each annotation term ("Name") based on Fisher´s Exact Test; "q-value" is the p-value corrected for multiplicity; "Hit type" defines the category of the curated pathway database

Supplementary Data 4 | Cell cluster marker genes sPD. Marker genes (HGNC symbols) identified for cell clusters NSC1 with subclusters NSC1a and NSC1b; NSC2 with subclusters NSC1a and NSC1b; Apoptotic NSCs; NCSCs with subcluster apoptotic NCSCs; Glial precursors; Immature neurons. Marker genes for apoptotic cell (AC) clusters were generated after cell cycle regression. Enrichment analysis based on marker genes of apoptotic NSCs and apoptotic NCSCs in curated pathways from Genomatix. Enriched terms with $p < 0.05$ are shown. P values were determined by one-sided Fisher's exact tests. FDR corrected p-values are represented by q-values. Column headings: "Network"; "Network id"; "GO-Term"; "GO-Term id"; "P-value"; "Adjusted p-value"; "# Genes (observed)"; "# Genes (expected)"; "# Genes (total)"; "List of observed genes"; "Gene ids"

Supplementary Data 5 | DEGs ($q < 0.05$) of different cell clusters from sPD patients. Column headings: HGNC "gene" symbol; "qval" p-value corrected for multiplicity; "fc" fold change of DEGs in sPD; "mean" normalized count levels; "coef sd" standard deviation for mean count levels

Supplementary Data 6 | Enriched PathwayStudio terms sPD. Enrichment analysis based on all DEGs of different cell clusters from sPD patients in curated pathways from the Pathway Studio Web (Elsevier). Enriched terms with $p < 0.05$ are shown. P values were determined by one-sided Fisher's exact tests. FDR corrected p-values are represented by q-values.
Column headings: "Name" denotes the annotation term; "Parent Folder" parent of the annotation term "Name" extracted from literature; "# of Entities" number of genes with a given annotation; "Expanded # of Entities" number of genes with a given annotation including associated members of functional protein classes; "Overlap" intersect of the number of genes from the input set with the number of genes from a given annotation ("Expanded # of Entities"); "Percent Overlap" proportion of overlapping genes from the input set in a given annotation ("Expanded # of Entities"); "Overlapping Entities" summarizes gene symbols from the input set with assigned annotation; "p-value" is the probability of overrepresentation calculated for each annotation term ("Name") based on Fisher´s Exact Test; "q-value" is the p-value corrected for multiplicity; "Hit type" defines the category of the curated pathway database

Supplementary Data 7 | Enriched KEGG terms sPD. Enrichment analysis based on all DEGs of different cell clusters from sPD patients using KEGG terms. Enriched terms with $p < 0.05$ are shown. P values were determined by one-sided hypergeometric tests. p-values corrected for multiplicity are represented by q-values.
Column headings: "ID" denotes the KEGG pathway identification number; "Description" denotes the annotation term; "GeneRatio" ratio of input genes that are annotated in a certain KEGG term; "BgRatio" ratio of all genes that are annotated in this KEGG term to genes that are annotated in all KEGG terms; "p-value" is the probability of overrepresentation calculated for each annotation term ("ID") based on Fisher´s Exact Test; "q-value" is the p-value corrected for multiplicity; "geneID" list of input genes (Entrez Gene ID) that are annotated in a certain KEGG term ; "Count" number of input genes that are annotated in a certain KEGG term.

Supplementary Data 8 | Enriched WikiPathways terms sPD. Enrichment analysis based on all DEGs of different cell clusters from sPD patients using WikiPathways (WP) terms. Enriched terms with $p < 0.05$ are shown. P values were determined by one-sided hypergeometric tests. p-values corrected for multiplicity are represented by q-values.
Column headings: "ID" denotes the WP pathway identification number; "Description" denotes the annotation term; "GeneRatio" ratio of input genes that are annotated in a certain WP term; "BgRatio" ratio of all genes that are annotated in this WP term to genes that are annotated in all WP terms; "p-value" is the probability of overrepresentation calculated for each annotation term ("ID") based on Fisher´s Exact Test; "q-value" is the p-value corrected for multiplicity; "geneID" list of input genes (Entrez Gene ID) that are annotated in a certain WP term ; "Count" number of input genes that are annotated in a certain WP term.

Supplementary Data 9 | Enriched PathwayStudio terms after thresholding sPD. Enrichment analysis based on DEGs ($| FC | > 20$ %; $q < 0.01$) of different cell clusters from sPD patients in curated pathways from the Pathway Studio Web (Elsevier). Enriched terms with $p < 0.05$ are

shown. P values were determined by one-sided Fisher's exact tests. FDR corrected p-values are represented by q-values.

Column headings: "Name" denotes the annotation term; "Parent Folder" parent of the annotation term "Name" extracted from literature; "# of Entities" number of genes with a given annotation; "Expanded # of Entities" number of genes with a given annotation including associated members of functional protein classes; "Overlap" intersect of the number of genes from the input set with the number of genes from a given annotation ("Expanded # of Entities"); "Percent Overlap" proportion of overlapping genes from the input set in a given annotation ("Expanded # of Entities"); "Overlapping Entities" summarizes gene symbols from the input set with assigned annotation; "p-value" is the probability of overrepresentation calculated for each annotation term ("Name") based on Fisher´s Exact Test; "q-value" is the p-value corrected for multiplicity; "Hit type" defines the category of the curated pathway database

Supplementary Data 10 | Pathways PD patients. Enrichment analysis based on DEGs from PD patients (published by [46]) in curated pathways from the Pathway Studio Web (Elsevier). Enriched terms with $p < 0.05$ are shown. P values were determined by one-sided Fisher's exact tests. FDR corrected p-values are represented by q-values.

Column headings: "Name" denotes the annotation term; "Parent Folder" parent of the annotation term "Name" extracted from literature; "# of Entities" number of genes with a given annotation; "Expanded # of Entities" number of genes with a given annotation including associated members of functional protein classes; "Overlap" intersect of the number of genes from the input set with the number of genes from a given annotation ("Expanded # of Entities"); "Percent Overlap" proportion of overlapping genes from the input set in a given annotation ("Expanded # of Entities"); "Overlapping Entities" summarizes gene symbols from the input set with assigned annotation; "p-value" is the probability of overrepresentation calculated for each annotation term ("Name") based on Fisher´s Exact Test; "q-value" is the p-value corrected for multiplicity; "Hit type" defines the category of the curated pathway database

Supplementary Data 11 | Cell cluster marker genes fPD. Marker genes (HGNC symbols) identified for *PINK1* ko cell clusters NSC1; Apoptotic NSCs; Immature neurons

Supplementary Data 12 | DEGs ($q < 0.05$) of different cell clusters from *PINK1* ko hNPCs. Column headings: HGNC "gene" symbol; "qval" p-value corrected for multiplicity; "fc" fold change of DEGs in sPD; "mean" normalized count levels; "coef sd" standard deviation for mean count levels

Supplementary Data 13 | Pathways fPD. Enrichment analysis based on all DEGs of NSC1 cluster from *PINK1* ko hNPCs in the category Cell Process of Pathway Studio Web (Elsevier). P values were determined by one-sided Fisher's exact tests. FDR corrected p-values are represented by q-values. Column headings as given for Supplementary Data 6. Enriched terms with $p < 0.05$ are shown. Terms related to intraflagellar transport are highlighted

Supplementary Data 14 | Detailed description of PD patients. Column headings: “study_ID” identifier for individuals in this study; “region” brain region analyzed for the respective individual; “gender” biological gender of the respective individual; “age” age in years of the respective individual at time of death; ”PMI” postmortem interval in hours of the respective individual; “LBD (Braak)” Braak stages to classify the degree of Lewy body disease (LBD) in the respective individuals; “AD (Braak & Braak)” Braak stages to classify the degree of Alzheimer’s disease (AD) in the respective individuals; “Aβ (Thal)” Thal phase based on a neuroanatomical hierarchy of amyloid β-protein (Aβ)-deposition in the respective individuals

Supplementary Data 15 | Data summary. Summary of archived data available for hiPSC lines, mice and human postmortem material on reasonable request
