# Supplementary material for: Primary cilia and SHH signaling impairments in human and mouse models of Parkinson’s disease
Source: Nat Commun. 2022 Aug 16;13:4819. doi: 10.1038/s41467-022-32229-9 (PMC9380673; doi:10.1038/s41467-022-32229-9)
Supplement: Supplementary file 3 — Description of Additional Supplementary Files [file 41467_2022_32229_MOESM3_ESM.pdf]

Cile ƴaƳe: Supplementary Data 1

DesǑℕiptioƴ: Detailed description of retroviral-hiPSC clones received from the ForIPS consortium [10]. Column headings: “study_ID_sample” identifier for hiPSC clones in this study; “study_ID_i ndividual” identifier for individuals in this study; “gender” biological gender of the respective individual; “age_biopsy_years” age in years of the respective individual at time of tissue biopsy; “years_of_illness” time in years between sPD diagnosis and tissue biopsy of the respective individual

Cile ƴaƳe: Supplementary Data 2

DesǑℕiptioƴ: CNVs in hiPSC clones. CNVs identified for all hiPSC clones and genes (HGNC symbols) within these regions. Column headings: “State”; “Sample ID”; “Chromosome”; “Start [bp]”; “End [bp]”; “Size [bp]”; “Copy numbers”; “Genes within these regions”

Cile ƴaƳe: Supplementary Data 3

DesǑℕiptioƴ: Pathways affected by CNVs. Enrichment analysis based on genes affected by CNVs in Ctrl or sPD hiPSC clones in curated pathways from the Pathway Studio Web (Elsevier). Enriched terms with $p < 0.05$ are shown for Ctrl or sPD clones. P values were determined by one-sided Fisher’s exact tests. FDR corrected p-values are represented by q-values.
Column headings: “Name” denotes the annotation term; “Parent Folder” parent of the annotation term “Name” extracted from literature; “# of Entities” number of genes with a given annotation; “Expanded # of Entities” number of genes with a given annotation including associated members of functional protein classes; “Overlap” intersect of the number of genes from the input set with the number of genes from a given annotation (“Expanded # of Entities”); “Percent Overlap” proportion of overlapping genes from the input set in a given annotation (“Expanded # of Entities”); “Overlapping Entities” summarizes gene symbols from the input set with assigned annotation; “p-value” is the probability of overrepresentation calculated for each annotation term (“Name”) based on Fisher´s Exact Test; “q-value” is the p-value corrected for multiplicity; “Hit type” defines the category of the curated pathway database

Cile ƴaƳe: Supplementary Data 4 | Cell cluster marker genes sPD. Marker genes (HGNC symbols) identified

for cell clusters NSC1 with subclusters NSC1a and NSC1b; NSC2 with subclusters NSC1a and NSC1b; Apoptotic NSCs; NCSCs with subcluster apoptotic NCSCs; Glial precursors; Immature neurons. Marker genes for apoptotic cell (AC) clusters were generated after cell cycle regression. Enrichment analysis based on marker genes of apoptotic NSCs and apoptotic NCSCs in curated pathways from Genomatix. Enriched terms with $p < 0.05$ are shown. P values were determined by one-sided Fisher’s exact tests. FDR corrected p-values are represented by q-values. Column headings: “Network”; “Network id”; “GO-Term”; “GO-Term id”; “P-value”; “Adjusted p-value”; “# Genes (observed)”; “# Genes (expected)”; “# Genes (total)”; “List of observed genes”; “Gene ids”

Cile ýaϒe: Supplementary Data 5

DesǑℕiptioý: DEGs (q < 0.05) of different cell clusters from sPD patients. Column headings: HGNC “gene” symbol; “qval” p-value corrected for multiplicity; “fc” fold change of DEGs in sPD; “mean” normalized count levels; “coef sd” standard deviation for mean count levels

Cile ýaϒe: Supplementary Data 6

DesǑℕiptioý: Enriched PathwayStudio terms sPD. Enrichment analysis based on all DEGs of different cell clusters from sPD patients in curated pathways from the Pathway Studio Web (Elsevier). Enriched terms with p < 0.05 are shown. P values were determined by one-sided Fisher’s exact tests. FDR corrected p-values are represented by q-values.

Column headings: “Name” denotes the annotation term; “Parent Folder” parent of the annotation term “Name” extracted from literature; “# of Entities” number of genes with a given annotation; “Expanded # of Entities” number of genes with a given annotation including associated members of functional protein classes; “Overlap” intersect of the number of genes from the input set with the number of genes from a given annotation (“Expanded # of Entities”); “Percent Overlap” proportion of overlapping genes from the input set in a given annotation (“Expanded # of Entities”); “Overlapping Entities” summarizes gene symbols from the input set with assigned annotation; “p-value” is the probability of overrepresentation calculated for each annotation term (“Name”) based on Fisher´s Exact Test; “q-value” is the p-value corrected for multiplicity; “Hit type” defines the category of the curated pathway database

Cile ýaϒe: Supplementary Data 7

DesǑℕiptioý: Enriched KEGG terms sPD. Enrichment analysis based on all DEGs of different cell clusters from sPD patients using KEGG terms. Enriched terms with p < 0.05 are shown. P values were determined by one-sided hypergeometric tests. p-values corrected for multiplicity are represented by q-values.

Column headings: “ID” denotes the KEGG pathway identification number; “Description” denotes the annotation term; “GeneRatio” ratio of input genes that are annotated in a certain KEGG term; “BgRatio” ratio of all genes that are annotated in this KEGG term to genes that are annotated in all KEGG terms; “p-value” is the probability of overrepresentation calculated for each annotation term (“ID”) based on Fisher´s Exact Test; “q-value” is the p-value corrected for multiplicity; “geneID” list of input genes (Entrez Gene ID) that are annotated in a certain KEGG term ; “Count” number of input genes that are annotated in a certain KEGG term.

Supplementary Data 8

DesǑℕiptioý: Enriched WikiPathways terms sPD. Enrichment analysis based on all DEGs of different cell clusters from sPD patients using WikiPathways (WP) terms. Enriched terms with p < 0.05 are shown. P values were determined by one-sided hypergeometric tests. p-values corrected for multiplicity are represented by q-values.

Column headings: “ID” denotes the WP pathway identification number; “Description” denotes the annotation term; “GeneRatio” ratio of input genes that are annotated in a certain WP term;

“BgRatio” ratio of all genes that are annotated in this WP term to genes that are annotated in all WP terms; “p-value” is the probability of overrepresentation calculated for each annotation term (“ID”) based on Fisher´s Exact Test; “q-value” is the p-value corrected for multiplicity; “geneID” list of input genes (Entrez Gene ID) that are annotated in a certain WP term ; “Count” number of input genes that are annotated in a certain WP term.

Cile ẏaϒe: Supplementary Data 9

DesǑiptioẏ: Enriched PathwayStudio terms after thresholding sPD. Enrichment analysis based on DEGs (| FC | > 20 %; q < 0.01) of different cell clusters from sPD patients in curated pathways from the Pathway Studio Web (Elsevier). Enriched terms with p < 0.05 are shown. P values were determined by one-sided Fisher’s exact tests. FDR corrected p-values are represented by q-values.

Column headings: “Name” denotes the annotation term; “Parent Folder” parent of the annotation term “Name” extracted from literature; “# of Entities” number of genes with a given annotation; “Expanded # of Entities” number of genes with a given annotation including associated members of functional protein classes; “Overlap” intersect of the number of genes from the input set with the number of genes from a given annotation (“Expanded # of Entities”); “Percent Overlap” proportion of overlapping genes from the input set in a given annotation (“Expanded # of Entities”); “Overlapping Entities” summarizes gene symbols from the input set with assigned annotation; “p-value” is the probability of overrepresentation calculated for each annotation term (“Name”) based on Fisher´s Exact Test; “q-value” is the p-value corrected for multiplicity; “Hit type” defines the category of the curated pathway database

Cile ẏaϒe: Supplementary Data 10

DesǑiptioẏ: Pathways PD patients. Enrichment analysis based on DEGs from PD patients (published by [46]) in curated pathways from the Pathway Studio Web (Elsevier). Enriched terms with p < 0.05 are shown. P values were determined by one-sided Fisher’s exact tests. FDR corrected p-values are represented by q-values.

Column headings: “Name” denotes the annotation term; “Parent Folder” parent of the annotation term “Name” extracted from literature; “# of Entities” number of genes with a given annotation; “Expanded # of Entities” number of genes with a given annotation including associated members of functional protein classes; “Overlap” intersect of the number of genes from the input set with the number of genes from a given annotation (“Expanded # of Entities”); “Percent Overlap” proportion of overlapping genes from the input set in a given annotation (“Expanded # of Entities”); “Overlapping Entities” summarizes gene symbols from the input set with assigned annotation; “p-value” is the probability of overrepresentation calculated for each annotation term (“Name”) based on Fisher´s Exact Test; “q-value” is the p-value corrected for multiplicity; “Hit type” defines the category of the curated pathway database

Cile ýaYe: Supplementary Data 11

DesǑiptioý: Cell cluster marker genes fPD. Marker genes (HGNC symbols) identified for *PINK1* ko cell clusters NSC1; Apoptotic NSCs; Immature neurons

Cile ýaYe: Supplementary Data 12

DesǑiptioý: DEGs (q < 0.05) of different cell clusters from *PINK1* ko hNPCs. Column headings: HGNC “gene” symbol; “qval” p-value corrected for multiplicity; “fc” fold change of DEGs in sPD; “mean” normalized count levels; “coef sd” standard deviation for mean count levels

Cile ýaYe: Supplementary Data 13

DesǑiptioý: Pathways fPD. Enrichment analysis based on all DEGs of NSC1 cluster from *PINK1* ko hNPCs in the category Cell Process of Pathway Studio Web (Elsevier). P values were determined by one-sided Fisher’s exact tests. FDR corrected p-values are represented by q-values. Column headings as given for Supplementary Data 6. Enriched terms with p < 0.05 are shown. Terms related to intraflagellar transport are highlighted

Cile ýaYe: Supplementary Data 14

DesǑiptioý: Detailed description of PD patients. Column headings: “study_ID” identifier for individuals in this study; “region” brain region analyzed for the respective individual; “gender” biological gender of the respective individual; “age” age in years of the respective individual at time of death; ”PMI” postmortem interval in hours of the respective individual; “LBD (Braak)” Braak stages to classify the degree of Lewy body disease (LBD) in the respective individuals; “AD (Braak & Braak)” Braak stages to classify the degree of Alzheimer’s disease (AD) in the respective individuals; “Aβ (Thal)” Thal phase based on a neuroanatomical hierarchy of amyloid β-protein (Aβ)-deposition in the respective individuals

Cile ýaYe: Supplementary Data 15

DesǑiptioý: Data summary. Summary of archived data available for hiPSC lines, mice and human postmortem material on reasonable request
