# Supplementary material for: Primary cilia and SHH signaling impairments in human and mouse models of Parkinson’s disease
Source: Nat Commun. 2022 Aug 16;13:4819. doi: 10.1038/s41467-022-32229-9 (PMC9380673; doi:10.1038/s41467-022-32229-9)
Supplement: Supplementary file 19 — Reporting Summary [file 41467_2022_32229_MOESM19_ESM.pdf]

nature portfolio

Corresponding author(s): Wolfgang Wurst
Last updated by author(s): Jun 17, 2022

Reporting Summary

Nature Portfolio wishes to improve the reproducibility of the work that we publish. This form provides structure for consistency and transparency in reporting. For further information on Nature Portfolio policies, see our Editorial Policies and the Editorial Policy Checklist.

Statistics

For all statistical analyses, confirm that the following items are present in the figure legend, table legend, main text, or Methods section.

n/a	Confirmed	
☐	☒	The exact sample size (n) for each experimental group/condition, given as a discrete number and unit of measurement
☐	☒	A statement on whether measurements were taken from distinct samples or whether the same sample was measured repeatedly
☐	☒	The statistical test(s) used AND whether they are one- or two-sided<br>*Only common tests should be described solely by name; describe more complex techniques in the Methods section.*
☐	☒	A description of all covariates tested
☐	☒	A description of any assumptions or corrections, such as tests of normality and adjustment for multiple comparisons
☐	☒	A full description of the statistical parameters including central tendency (e.g. means) or other basic estimates (e.g. regression coefficient) AND variation (e.g. standard deviation) or associated estimates of uncertainty (e.g. confidence intervals)
☐	☒	For null hypothesis testing, the test statistic (e.g. F, t, r) with confidence intervals, effect sizes, degrees of freedom and P value noted<br>*Give P values as exact values whenever suitable.*
☒	☐	For Bayesian analysis, information on the choice of priors and Markov chain Monte Carlo settings
☒	☐	For hierarchical and complex designs, identification of the appropriate level for tests and full reporting of outcomes
☒	☐	Estimates of effect sizes (e.g. Cohen's d, Pearson's r), indicating how they were calculated

Our web collection on statistics for biologists contains articles on many of the points above.

Software and code

Policy information about availability of computer code

Data collection	No software was used for data collection.
Data analysis	Clustering, quality control, and SNP calling was done using GenomeStudio 2.0 (Illumina) and CNVs on autosomes were detected using cnvPartition v3.2.1 (Illumina). SNP files were converted to vcf files using PLINK 2.00 alpha. SNPs were converted to the GRCh38 forward strand using a script developed by Robertson and Wrayner (https://www.well.ox.ac.uk/~wrayner/strand/; 12.11.2019). A custom-made perl script was used to extract exonic SNPs using exon boundaries obtained from BioMart.<br>Running in python version 3.6.7 and R version 3.5.1:<br>Sequencing data was processed using Cell Ranger version 2.1.1. Read files were aligned against hg38 from Ensembl release 94 using default parameters. Spliced and unspliced counts for RNA velocity analysis were called using velocyto version 0.17.7 and samtools version 1.7.<br>Demuxlet (retrieved 17th July 2018) and vcf tools version 0.1.15 were used to demultiplex the pooled sequencing data.<br>UMI count, unspliced, and spliced data were processed using Scanpy version 1.4.3 commit 0075c62 and ScVelo version 0.1.24 commit e45a65a.<br>Normalization and batch correction was performed in Scran verison 1.10.2 and mnnpy version 0.1.9.5.<br>Clustering was performed using the louvain package (version 0.6.1) and visualization was done via umap (version 0.3.9).<br>Compositional changes within clusters between Ctrl and sPD conditions were assesed using scCODA version 0.1.7.<br>Gene set enrichment was performed in g:profiler (version 1.2.0).<br>Enrichment in the curated pathway categories “Cell Process” and “Signal Processing” was performed using Pathway Studio software (Elsevier) version 12.4.0.5 and q-values were calculated using the fdrtool v1.2.16 R package.<br>Running in R version 4.0.5 and RStudio Desktop 1.2.1335:<br>Enriched gene sets of the category curated canonical pathways (C2.CP collection of the Molecular Signatures Database v7.5.1), KEGG and WikiPathway pathways were analyzed using the R package clusterProfiler version 4.2.2.<br>Heatmap were generated by using the R package gplots v3.1.1.<br>Vulcano plots were generated by using the R package EnhancedVolcano version 1.12.0.

In silico prediction of transcription factor binding sites in promoter sequences was performed by using the MatInspector program v8.4.1 (Genomatix) and the Matrix Family Library Version 11.1 (February 2019).
Distribution plots were generated and compared using the R package "sm: Smoothing Methods for Nonparametric Regression and Density Estimation" version 2.2-5.6.
Venn diagrams were generated using the R package "VennDiagram" Version 1.6.20.
Linear mixed effects model were fit using the lmer function (R package "lme4" Version 1.1-26), p values were calculated using the Anova function (R package "car" Version 3.0-10).
All other visualizations and statistical analyses were performed using GraphPad Prism 6.
Neurolucida version 2019.2.1 was used for cilia length quantification.
ImageJ version 1.53c was used for cilia length quantification and image adjustments.
Image Lab 6.1 was used for western blot quantification.
High content screening was performed using HCS Studio 2.0.
Wave 2.6.1 was used for visualizing and exporting Seahorse XF data.
Analysis code is freely available at [https://github.com/theislab/ipsc_ipd_analysis][https://doi.org/10.5281/zenodo.6656506].

For manuscripts utilizing custom algorithms or software that are central to the research but not yet described in published literature, software must be made available to editors and reviewers. We strongly encourage code deposition in a community repository (e.g. GitHub). See the Nature Portfolio guidelines for submitting code & software for further information.

Data

Policy information about availability of data

All manuscripts must include a data availability statement. This statement should provide the following information, where applicable:

- Accession codes, unique identifiers, or web links for publicly available datasets
- A description of any restrictions on data availability
- For clinical datasets or third party data, please ensure that the statement adheres to our policy

All data produced in this study (Supplementary Data 15) are archived internally. Source Data are provided as a Source Data file which is also deposited in Zenodo as record 6677636 [https://doi.org/10.5281/zenodo.6677636]. The scRNA-seq data were deposited in the NCBI Gene Expression Omnibus (GEO) under accession number GEO: GSE176160 [https://www.ncbi.nlm.nih.gov/geo/query/acc.cgi?acc=GSE176160]. For further requests please contact the corresponding author (W.W.). Following databases were used within this study: Pathway Studio Web (Elsevier) Mammal Database Version 12.4.0.5 [https://www.pathwaystudio.com] (downloaded on 03.2022), KEGG pathways [https://www.genome.jp/kegg/pathway.html] (downloaded on 03.2022), WikiPathway pathways [https://www.wikipathways.org/index.php/WikiPathways] (downloaded on 03.2022), C2.CP collection of the Molecular Signatures Database Version 7.5.1 [https://www.gsea-msigdb.org/gsea/msigdb/] (downloaded on 03.2022), BioMart (https://www.ensembl.org/info/data/biomart/index.html#biomartdoc] (downloaded on 11.2019), Ensembl hg38 release 94 [http://oct2018.archive.ensembl.org/Homo_sapiens/Info/Index] (downloaded on 02.2019), Matrix Family Library Version 11.1 [https://www.genomatix.de/online_help/help_gems/mat_lib_111.html] (downloaded on 02.2019).

Field-specific reporting

Please select the one below that is the best fit for your research. If you are not sure, read the appropriate sections before making your selection.

☒ Life sciences ☐ Behavioural & social sciences ☐ Ecological, evolutionary & environmental sciences

For a reference copy of the document with all sections, see nature.com/documents/nr-reporting-summary-flat.pdf

Life sciences study design

All studies must disclose on these points even when the disclosure is negative.

Sample size	No explicit calculations were performed to determine sample size since all (at this time) available and well characterized hiPSC lines provided by the ForIPS consortium were used for analysis. Thus, we used the largest available sample size possible. The sample size was sufficient to perform reliable data analysis. For all other experiments, sample size was estimated based on previous experience in which 4 or more independent samples per group were sufficient to detect significant differences.
Data exclusions	Only transcriptome data from cells called as singlets by demuxlet were used for further processing. Additionally, cells with more than 45,000 counts, with fewer than 1,000 genes expressed, and with 15% or more reads aligned to mitochondrial genes were filtered out.<br>For all other experiments, outliers identified by the ROUT method (Q = 0.5%; GraphPad) were removed for statistical analysis.
Replication	For validation of the single cell RNA sequencing data, mean values from three independent repetitions per cell line are shown and were used for statistical analysis. These experiments validated the findings derived from the single cell RNA sequencing.<br>Similarly for quantification of mitochondrial function, quantification of neurons/dopaminergic neurons, quantification of dopaminergic neurites, quantification of primary cilia and SHH signaling as well as rescue experiments, mean values from three independent repetitions per cell line are shown and were used for statistical analysis. All experiments were successfully repeated three times. This takes not into account the repetitions that had to be discarded due to technical issues (e.g. detection problems of bands on western blots).<br>For experiments using unique biological material (human and mouse brain slides), material from multiple individuals as well as multiple slides per individual were used.
Randomization	Individuals were phenotypically examined by a clinician experienced with neurological diseases. Parkinsons`s disease patients were diagnosed by board-examined movement disorder specialists according to consensus criteria of the German Society of Neurology, which are similar to the UK PD Society Brain Bank criteria for diagnosis of PD. All Parkinsons`s disease patients were characterized for the absence of known PD-causing familial mutations (PARK 1-18). For the hiPSC clones, this has been described by Popp et al. 2018 [https://doi.org/10.1038/s41598-018-35506-0].

Pink1 ko mice were distinguished from wild type littermates using a PCR analysis as described by Glasl et al. 2012 [https://doi.org/10.1016/j.expneurol.2012.01.002].
PINK1 ko hiPSCs were distinguished from their isogenic Ctrl using a PCR analysis and sequencing as described by Bus et al. 2020 [https://doi.org/10.1016/j.isci.2020.101797].
The lesion site in brain slides from 6-OHDA treated mice was identified by performing a immunostaining for TH after the quantification of primary cilia was completed.

Blinding	Investigators were blinded for data processing and exclusions.

Reporting for specific materials, systems and methods

We require information from authors about some types of materials, experimental systems and methods used in many studies. Here, indicate whether each material, system or method listed is relevant to your study. If you are not sure if a list item applies to your research, read the appropriate section before selecting a response.

Materials & experimental systems

n/a	Involved in the study
☐	☒ Antibodies
☐	☒ Eukaryotic cell lines
☒	☐ Palaeontology and archaeology
☐	☒ Animals and other organisms
☐	☒ Human research participants
☒	☐ Clinical data
☒	☐ Dual use research of concern

Methods

n/a	Involved in the study
☒	☐ ChIP-seq
☒	☐ Flow cytometry
☒	☐ MRI-based neuroimaging

Antibodies

Antibodies used	AC-TUB (T6793, Sigma-Aldrich; 1:1000), ARL13B (17711-1-AP, Proteintech; 1:500, Lot Nr. 00087629), GFAP (MAB360, Millipore; 1:250, Lot Nr. 3465434), GLI3 (AF3690, R&D; 1:100, YLD0321072), NANOG (AF1997, R&D Systems; 1:200, Lot Nr. KKJ0616121), NES (Ma1110, Thermo Fisher Scientific; 1:250), PAX6 (Ab78545, Abcam; 1:200), PITX3 (38-2850, Invitrogen; 1:300, Lot Nr. QD215204), POU5F1 (2840, Cell Signaling; 1:500), RBFOX3 (ab104224, Abcam; 1:800, Lot Nr. GR3408621-2), SLC1A3 (NB100-1869, Novus Biologicals; 1:250, Lot Nr. F-2), SMO (sc166685, Santa Cruz; 1:500), SOX1 (Ab87775, Abcam; 1:500, Lot Nr. GR3298895-2), SOX2 (sc17320, Santa Cruz; 1:500), TH (P40101, PelFreez; 1:600, Lot Nr. ajo520p), TUBB3 (T5076, Sigma-Aldrich; 1:1000, Lot Nr. 0000134483), Adcy3 (PA5-35382, Invitrogen; 1:500, Lot Nr. VE2997041), H1-0 (ab11079, Abcam; 1 µg/ml, Lot Nr. GR69708-1), Chat (AB144P, Millipore; 1:200, Lot Nr. 3491643), Rbfox3 (MAB377, Millipore; 1:200), ACTB (ABO145-200, OriGene; 1:2,000, Lot Nr. 0145131020), PCNA (ab29, Abcam; 1 µg/ml, Lot Nr. GR201287-23), donkey-anti-goat IgG Alexa 488 (A11055, Thermo Fisher Scientific; 1:500), donkey-anti-mouse IgG Alexa 488 (A21202, Thermo Fisher Scientific; 1:500), donkey-anti-goat IgG Alexa 594 (A11058, Thermo Fisher Scientific; 1:500), donkey-anti-mouse IgG Alexa 594 (A21203, Thermo Fisher Scientific; 1:500), donkey-anti-rabbit IgG Alexa 488 (A21206, Thermo Fisher Scientific; 1:500), donkey-anti-rabbit IgG Alexa 594 (A21207, Thermo Fisher Scientific; 1:500), rabbit-anti-mouse IgG peroxidase (GTX213112-01, GeneTex; 1:10,000), rabbit-anti-goat IgG peroxidase (305-035-003, Dianova; 1:10,000), Biotin-SP-conjugated goat anti-rabbit IgG (111-065-003, Dianova; 1:250).
Validation	AC-TUB (T6793; Sigma-Aldrich; 1:1000; validation*: applications: Dot blot, ELISA, EM, ICC, Radio, WB; species reactivity: e.g. Bovine, Green monkey, Chicken, Human, Hamster, Mouse, Rat, Pig, Xenopus; citations: 1340x - e.g. Vuolo et al., 2018; Park et al., 2021; Sillibourne et al., 2013; Ohata et al., 2015)<br>ARL13B (17711-1-AP, Proteintech; 1:500; validation*: applications: ELISA, IF, IHC, IP, WB; species reactivity: Dog, Rat, Mouse, Human, citations: 527x cited - e.g. Ki et al., 2020; Bae et al., 2019; Wang et al., 2018)<br>GFAP (MAB360; Millipore; 1:250; validation*: applications: ICC, IHC, IHC-P, WB; species reactivity: e.g. Bovine, Chicken, Human, Mouse, Rabbit, Rat, Pig, citations: 975x - e.g. Cherry et al., 2018; Wang et al., 2019; Lu et al., 2017)<br>GLI3 (AF3690, R&D; 1:100; validation*: applications: e.g. ChiP, ICC, WB; species reactivity: Human, Mouse; citations: 113x - e.g. Hwang et al., 2021; Freisleben et al., 2020; Nam et al., 2019)<br>NANOG (AF1997, R&D Systems; 1:200; validation*: applications: e.g. ChiP; ICC, Wb; species reactivity: Human, Macaca, Mouse citations: 319x - e.g. Lehtonen et al., 2018; Shafa et al., 2018; Yahata et al., 2017)<br>NES (Ma1-110, Invitrogen; 1:250; validation*: applications: e.g. FACS, ICC, IF, IHC, IHC-P, WB; species reactivity: Human, Mouse, citations: 26x - e.g. Sharma et al., 2020; Liu et al., 2022; Kimura et al., 2021)<br>PAX6 (Ab78545, Abcam; 1:200; validation*: applications: e.g. ELISA, ICC, IHC; species reactivity: Chicken, Human, Mouse, Rat, citations: 38x - e.g. Penna et al., 2021; Sawada et al., 2029; Peng et al., 2018)<br>PITX3 (38-2850, Invitrogen; 1:300; validation*: applications: e.g. ICC, IF, IHC; species reactivity: e.g. Mouse, Human, Rat, citations: 20x - e.g. Kim et al., 2019; Jovanovic et al., 2018; Jiang et al., 2015)<br>POU5F1 (2840S, Cell Signaling; 1:500; validation*: applications: e.g. IF, WB; species reactivity: e.g. Human, Mouse; citations: 153x - e.g. Hazim et el., 2017; Swaroop et al., 2018; Saini et al., 2017)<br>RBFOX3 (ab104224, Abcam; 1:800; validation*: applications: e.g. ICC, IHC, WB; species reactivity: e.g. Bovine, Dog, Goat, Cat, Chicken, Human Mouse, Rat, Pig, citations: 423x- e.g. Li et al., 2022; Szebényi et al. 2021; Zhu et al., 2016)<br>SLC1A3 (NB100-1869, Novus Biologicals; 1:250; validation*: applications: e.g. ELISA, ICC, IHC, WB; species reactivity: e.g. Human, Mouse, Rat; citations: 18x - e.g. Rostami et al., 2020; Sandau et al., 2022; Reyes-Aguirre et al., 2016)<br>SMO (sc166685, Santa Cruz; 1:500; validation*: applications: e.g. ELISA, IF, IP, WB; species reactivity: e.g. Human, Mouse, Rat; citations: 59x - e.g. Bosakova et al., 2019; Kozielewicz et al., 2020; He et al., 2017)<br>SOX1 (Ab87775, Abcam; 1:500; validation*: applications: e.g. ICC, IHC, WB; species reactivity: e.g. Dog, Human, Mouse, Rabbit, Rat; citations: 20x - e.g. Hor et al., 2018; Aneichyk et al., 2018; Lei et al., 2019)

SOX2 (sc17320, Santa Cruz; 1:500; validation*: applications: e.g. IHC, IF; species reactivity: e.g. Human, Mouse, Rat; citations: - e.g. 766x Gao et al., 2019; Marchetto et al., 2019)
TH (P40101, PelFreez; 1:600; validation*: applications: e.g. ELISA, IF, IHC, WB; species reactivity: e.g. Mouse, Human (Mammals); citations: - e.g. Bordone et al., 2021; Park et al., 2020; Jarras et al., 2020)
TUBB3 (T5076, Sigma-Aldrich; 1:1000; validation*: applications: e.g. ELISA; ICC, IHC, WB; species reactivity: e.g. Bovine, Human, Mouse, Rat, Pig; citations: 73x - e.g. Nardone et al., 2017; Ichinose et al., 2021; Sousa et al, 2020)
Adcy3 (PA5-35382, Invitrogen; 1:500; validation*: applications: e.g. IF, IHC, WB; species reactivity: e.g. Human, Mouse, Rat; citations: 4x - e.g. Mifsud et al., 2021; Xie et al., 2021; Zhang et al., 2019)
H1-0 (ab11079, Abcam; 1 µg/ml; validation*: applications: e.g. IHC, WB; species reactivity: e.g. Human, Mouse, Rat, Xenopus; citations: 29x - e.g. Meseure et al., 2020; Serna-Pujol et al., 2022; Falbo et al., 2020)
Chat (AB144P, Millipore; 1:200; validation*: applications: e.g. ICC, IHC, WB; species reactivity: e.g. Pig, Chicken, Human Mouse, Rat; citations: 1647x - e.g. Lai et al., 2018; Allodi et al., 2016; Ryczko et al., 2016)
ACTB (ABO145-200, OriGene; 1:2,000; validation*: applications: e.g. IF, WB; species reactivity: e.g. Cat, Human, Monkey, Mouse, Rat; citations: e.g. Ishizuka et al., 2020)
PCNA (ab29, Abcam; 1 µg/ml; validation*: applications: e.g. FACS, IHC, IP, WB; species reactivity: e.g. Bovien Chicken, Human, Mouse, Rat, Pig; citations: 616x - e.g. Zuo et al., 2021; Hu et al., 2020; Matsuno et al., 2020)
RBFOX3 (MAB377, Millipore; 1:200; validation*: applications: e.g. FACS, IHC, IP, IF, WB; species reactivity: e.g. Chicken, Human, Mouse, Rat; citations: 5123x - e.g. Noristani et al., 2022; Qin et al., 2021; Miyake et al., 2021)

*according to manufacturers' website and/or www.citeab.com

Eukaryotic cell lines

Policy information about cell lines

Cell line source(s)

sPD and Ctrl hiPSC lines were established, characterized and provided by the ForIPS consortium (University Hospital Erlangen) (Supplementary Data 1).
The TALEN induced PINK1 knockout (ko) line and the isogenic Ctrl were established, characterized and provided by Julia Fitzgerald (University of Tübingen).

Authentication

Cell lines were authenticatedin the ForIPS consortium by analyzing 20 polymorphic markers located on 15 different chromosomes to exclude handling errors. This has been recently published by Popp et al. 2018 [https://doi.org/10.1038/s41598-018-35506-0].
PINK1 ko hiPSCs were authenticated and distinguished from their isogenic Ctrl using a PCR analysis and sequencing as described by Bus et al. 2020 [https://doi.org/10.1016/j.isci.2020.101797].
At the end of experiments, karyotyping was repeated to ensure the integrity of all cell lines.

Mycoplasma contamination

All cell lines were tested negative for mycoplasma contamination.

Commonly misidentified lines
(See ICLAC register)

No misidentified cell line was used.

Animals and other organisms

Policy information about studies involving animals; ARRIVE guidelines recommended for reporting animal research

Laboratory animals

12.5 ± 1.5 months old male homozygous Pink1-/- mice and wild type littermate Pink1+/+ Ctrl animals were analyzed. Mice have been bred on a C57BL/6J genetic background.
For the analysis of 6-hydroxydopamine-HCl (6-OHDA-HCl) lesion mice, three 3-4-month-old male homozygous B6.Cg-Tg(Gfapcre)77.6Mvs/2J (Jackson Laboratories) littermates (breeding of heterozygous parents) were analyzed.

Wild animals

Studie did not involve wild animals.

Field-collected samples

Studie did not involve field-collected samples.

Ethics oversight

All animal protocols and procedures were conducted with the approval for the ethical treatment of animals by the responsible animal welfare authority of the Regierung von Oberbayern (Government of Upper Bavaria) and considering the 3R principle.

Note that full information on the approval of the study protocol must also be provided in the manuscript.

Human research participants

Policy information about studies involving human research participants

Population characteristics

The relevant population characteristics of the human research participants including age [in years], gender [male = m; female = f], the post-mortem interval [in hours] and diagnosis informations ["LBD (Braak)" Braak stages to classify the degree of Lewy body disease (LBD) in the respective individuals; "AD (Braak & Braak)" Braak stages to classify the degree of Alzheimer's disease (AD) in the respective individuals; "Aβ (Thal)" Thal phase based on a neuroanatomical hierarchy of amyloid β-protein (Aβ)-deposition in the respective individuals] are also summarized in Supplementary Data 14.

study_id [region; gender; age_years; PMI_hours; LBD (Braak); AD (Braak & Braak); Aβ (Thal)]
GA 2019/20 PD-1 [anterior striatum; m; 64; 34-58; 6; II; 0]
GA 2019/20 PD-2 [anterior striatum; m; 79; 49; 6; II; 0-1]

GA 2019/20 PD-3 [anterior striatum; m; 74; 42; 6; II; 0]
GA 2019/20 PD-4 [anterior striatum; f; 79; < 37; 6; II; 0]
GA 2019/20 PD-5 [anterior striatum; f; 76; 19; 6; I; 1]
GA 2019/20 PD-6 [anterior striatum; m; 78; 48; 6; II; 0]
GA 2019/20 Ctrl-1 [anterior striatum; f; 59; 12; 0; I; 1]
GA 2019/20 Ctrl-2 [anterior striatum; m; 62; 20; 0; I; 0]
GA 2019/20 Ctrl-3 [anterior striatum; m; 71; 17; 0; II; 0]
GA 2019/20 Ctrl-4 [anterior striatum; m; 61; 24; 0; I; 0]
GA 2019/20 Ctrl-5 [anterior striatum; f; 63; 14; 0; I; 1]
GA 2019/20 Ctrl-6 [anterior striatum; m; 71; 25; 0; I; 0]
GA 2019/20 PD-1 [occipital cortex; m; 64; 34-58; 6; II; 0]
GA 2019/20 PD-2 [occipital cortex; m; 79; 49; 6; II; 0-1]
GA 2019/20 PD-3 [occipital cortex; m; 74; 42; 6; II; 0]
GA 2019/20 PD-4 [occipital cortex; f; 79; < 37; 6; II; 0]
GA 2019/20 PD-5 [occipital cortex; f; 76; 19; 6; I; 1]
GA 2019/20 PD-6 [occipital cortex; m; 78; 48; 6; II; 0]
GA 2019/20 Ctrl-1 [occipital cortex; f; 59; 12; 0; I; 1]
GA 2019/20 Ctrl-2 [occipital cortex; m; 62; 20; 0; I; 0]
GA 2019/20 Ctrl-3 [occipital cortex; m; 71; 17; 0; II; 0]
GA 2019/20 Ctrl-4 [occipital cortex; m; 61; 24; 0; I; 0]
GA 2019/20 Ctrl-7 [occipital cortex; f; 64; 33; 0; I; 0]
GA 2019/20 Ctrl-8 [occipital cortex; f; 60; 15; 0; I; 0]

Recruitment

Individuals were phenotypically examined by a clinician experienced with neurological diseases. Parkinsons`s disease patients were diagnosed by board-examined movement disorder specialists according to consensus criteria of the German Society of Neurology, which are similar to the UK PD Society Brain Bank criteria for diagnosis of PD.
(1) Patients with a known familial form were excluded.
(2) Patients were age >60, with a defined LBD(Braak) stage of 5-6, a AD(Braak & Braak) stage of <3 and a Aβ (Thal) phase of <2.
(3) Control individuals were age >60, with a defined LBD(Braak) stage of 0, a AD(Braak & Braak) stage of <3 and a Aβ (Thal) phase of <2.
(4) Post-mortem interval was as short as possible.
(5) FFPE tissue of the anterior striatum and/or the occipital cortex was available.

Ethics oversight

Ethical approval for the work with human postmortem material was obtained from the Institutional Review Board of TU Munich, Munich, Germany (#419/19 S-SR). All donors or authorized relatives had provided written informed consent. Approved patient identifiers were used to conceal patient identity. Tissue from the anterior striatum and the occipital cortex was obtained from the Neurobiobank Munich (NBM) (www.en.neuropathologie.med.uni-muenchen.de/neurobiobank/neurobiobank/index.html).

Note that full information on the approval of the study protocol must also be provided in the manuscript.
